# Impact of prior CKD management in a renal care network on early outcomes in incident dialysis patients: a prospective observational study

**DOI:** 10.1186/1471-2369-14-41

**Published:** 2013-02-20

**Authors:** Nicolas Rognant, Eric Alamartine, Jean Claude Aldigier, Christian Combe, Benoit Vendrely, Patrice Deteix, Pascal Cluzel, Laurent Juillard, François Vrtovsnik, Christelle Maurice, Sophie Fave, Maurice Laville

**Affiliations:** 1 Département de Néphrologie, Hôpital Edouard Herriot, Hospices Civils de Lyon, Pavillon P 5 place d’Arsonval, Lyon Cedex 03 F-69437, France; 2 2 Université de Lyon, Lyon F-69373, France; 3 Réseau TIRCEL, Hôpital Edouard Herriot, Lyon Cedex 03, F-69437, France; 4 Service de Néphrologie, Hôpital Nord, Saint Etienne, F-42055, France; 5 Service de Néphrologie, Hôpital Dupuytren, Limoges Cedex, F-87042, France; 6 Service de Néphrologie, Transplantation, Dialyse, Centre Hospitalo-Universitaire de Bordeaux, Bordeaux, F-33000, France; 7 Université Victor Segalen, Bordeaux, F-33000, France; 8 Service de Néphrologie, Hôpital Gabriel Montpied, F-63003, Clermont Ferrand Cedex 1, France; 9 AURA Auvergne, Chamalières, F-63400, France; 10 Service de Néphrologie, Hôpital Bichat, Paris Cedex 18, F-75877, France; 11 Pôle IMER, Hospices Civils de Lyon, et EA Santé-Individus-Société, Université de Lyon, Lyon, F-69000, France

**Keywords:** Chronic kidney disease, Multidisciplinary intervention, Renal cares network, Cardiovascular events, CKD progression

## Abstract

**Background:**

Effective therapeutic strategies are available to prevent adverse outcomes in patients with chronic kidney disease (CKD) but their clinical results are hindered by unplanned implementation. Coordination of care emerges as a suitable way to improve patient outcomes. In this study, we evaluated the effect of planned and coordinated patient management within a dedicated renal care network comparatively to standard renal care delivered in nephrology departments of teaching hospitals.

**Methods:**

This observational matched cohort study included 40 patients with CKD stage 4–5 in the network group as compared with a control group of 120 patients matched for age, sex and diabetic status. Main outcome was a composite endpoint of death from cardiovascular cause and cardiovascular events during the first year after dialysis initiation.

**Results:**

There was no difference between the two groups neither for the primary outcome (40% vs 41%) nor for the occurrence of death from cardiovascular cause or cardiovascular events. Whereas the proportion of patients requiring at least one hospitalization was identical (83.3% vs 75%), network patients experienced less individual hospitalizations than control patients (2.3±2.0 vs 1.6±1.7) during the year before dialysis start. Patients of the network group had a slower renal function decline (7.7±2.5 vs 4.9±1.1 ml/min/1,73m^2^ per year; p=0.04).

**Conclusions:**

In this limited series of patients, we were unable to demonstrate a significant impact of the coordinated renal care provided in the network on early cardiovascular events in incident dialysis patients. However, during the predialysis period, there were less hospitalizations and a slower slope of renal function decrease.

## Background

Chronic Kidney Disease (CKD) is a growing concern in developed and developing countries. The continuous increase in the number of prevalent End-Stage Renal Disease (ESRD) patients led to an increased burden of care for patients and of expenses for the health care system [1]. A critical issue is the identification of patients with the highest risk of adverse outcomes, using currently available markers [2]. Another major issue is the most efficient way to deliver appropriate care to these patients.

Although efficient therapeutic interventions to prevent CKD progression exist since almost two decades, they seem to have had only limited impact on ESRD incidence during this period [3,4]. This lack of clear benefit is likely due to suboptimal cares, which can be observed both when the patient is followed by the general practitioner (GP) and/or the nephrologist [5-7].

Uncoordinated cares, the characteristics of health environment and some degree of therapeutic inertia related to physician’s behaviour are suspected to play a key role in poor therapeutic efficiency [8,9]. This has led to questions about the way to deliver renal care more efficiently. Multidisciplinary renal clinics (MDRC) [10] and renal care networks [11] both offer an integrative way to provide optimal renal cares. In patients with diabetes mellitus, studies have found positive effects of this type of care delivery on patient’s outcomes [12,13]. In CKD patients, several studies have also found some positive effects of MDRC comparatively to standard care on renal outcomes [14-18]. Presently, the best way to organize those multidisciplinary renal clinics is, however, not standardized. To our knowledge, there is only one experience reported concerning the effects of renal care networks [11]. We therefore undertook a prospective matched cohort study in order to assess the effects of prior management by a renal care network on early mortality and outcomes in incident patients on dialysis.

## Methods

The study was conducted in accordance with the principles of the declaration of Helsinki. The local ethical committee (*Comité de protection des personnes SUD-EST IV*, Lyon, France) approved the study protocol. The committee did not recommend obtaining informed consent from patients enrolled in this study because the data were analysed anonymously. This was a multicenter, prospective, matched-cohort study. The exposed patients were followed in the TIRCEL network (*Traitement de l’Insuffisance Rénale Chronique en rhônE-aLpes*), which is a coordinated care network dedicated to CKD patients in the *Rhône-Alpes* area. The management of patients in the network has been fully described elsewhere [19]. Briefly, there were standardized protocols for monitoring the patient’s clinical and biological status. The frequency of monitoring was based on the level of severity of CKD. To avoid losing sight of patients, they were contacted by telephone by members of the coordination of the network when they were not attended to a scheduled consultation. Educational sessions were proposed to the patients on a voluntary basis. The educational sessions and materials were built according to the French guidelines on patient’s education. Some cardiovascular risk factors, like Blood Pressure (BP) control and how to reach optimal blood level of cholesterol were specifically addressed during the educational sessions. At 12/31/2011, the network included 985 patients with 22.6% stage 4–5 CKD patients. The patients of the control group were all followed up in the nephrology department of one of the following hospitals which are all teaching hospitals located outside of the area covered by the network: Saint Etienne, Bordeaux, Limoges, Clermont Ferrand and Paris-Bichat. Those patients underwent standard care, which included periodic visits, and biological assays with frequency based on the level of glomerular filtration rate (GFR). A dedicated nurse rather than the physician gave information and preparation for dialysis.

**Figure 1:**
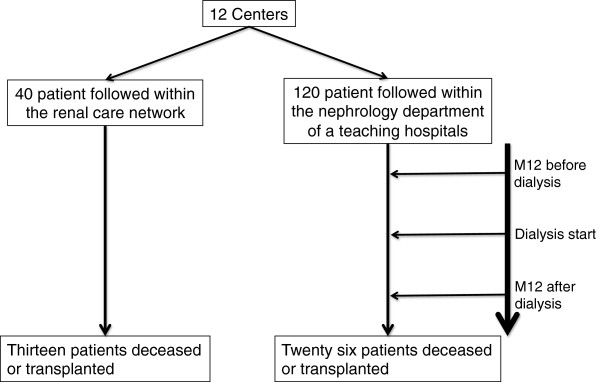
Flow-chart of the study.

All patients aged over 18 years, benefiting from social insurance and who started maintenance dialysis between January 2004 and August 2009 could be included. We chose the period just around the start of dialysis because dialysis patients have the greatest mortality rate during the first year of dialysis treatment. Moreover, suboptimal care during the period just before the start of dialysis can have serious consequences for patients with stage 4–5 CKD and we assumed that there is a potential for improving the management of patients during this period. Finally only patients who actually started dialysis treatment were included. During the study period, all of the 40 network patients who started dialysis were included in the analysis. Each network patient was individually matched for age, sex, diabetic status and dialysis start time with three randomly chosen control patients coming from 3 different nephrology departments (Figure 1).

### Data collection

Patient data were recorded retrospectively from 12 months before dialysis then prospectively for 12 months after the beginning of dialysis. The data were collected from either electronic or written medical records. All the data from medical visits and biological measurements, including at dialysis start, were collected over the pre-dialysis year. Over the first dialysis year, all events occurring within and between dialysis sessions were recorded. Estimated GFR (eGFR) was calculated by using simplified MDRD equation [20]. Measurement of BP was made in sitting position at rest during physician visit.

### Endpoints

The primary endpoint was the occurrence of cardiovascular events or death from cardiovascular cause during the first year on dialysis. Cardiovascular events were defined as one of the following events: myocardial infarction, congestive heart failure, ischemic or haemorrhagic stroke, acute lower limb ischemia, lower limb amputation.

The secondary endpoints were death from any cause and hospitalizations during the first year on dialysis. During the year before entering dialysis, we also studied the following events:

-Number of hospitalizations and slope of eGFR decline;

-Frequency of medical and dietetic visits and of monitoring of biological relevant parameters

-Prescription of blockers of renin-angiotensin system (RAS), beta-blockers, antiplatelet agents, and lipid-lowering drugs; rate of patients reaching target values for BP and the value of some relevant biological parameters

-Individual and dedicated information on dialysis treatments received by patients, rate of emergency first dialysis, rate of initial use of sustainable dialysis access, registration on transplantation waiting list.

### Statistical analysis

Characteristics of our study samples were compared to national data by one-sample Student t-tests (for means) or one-sample z-tests (for proportions). Variables were presented as mean, standard deviation for quantitative variables and as percentage for qualitative variables. Survival was estimated by the product-limit procedure of Kaplan Meier, and survival curves were compared with the log rank test. Biological or clinical parameters were compared using conditional logistic regression [21]. The calculation of the samples size, with α=0.05 and 1-β=0.8 were based on the following assumptions: risk of one year mortality or cardiovascular events of 20% with the follow up in the network allowing to decrease this risk to 5%. We found that 38 patients had to be included in the network group and 114 in the control group. Assuming a proportion of patient lost of follow up of almost 5%, we included 40 patients in the network group and 120 in the control group.

The GFR annual slope was computed with all values available between 12 months before dialysis and dialysis starting date. This slope was compared between the network group and the control group with a mixed model accounting for matching [22,23]. Using regression equations, we defined the theoretical value for entering dialysis. Only parameters with less than 15% of missing data were considered. Statistical analysis was performed using SAS 9.2 software (SAS Institute Inc., North Carolina, USA) and the threshold of 0.05 was considered as statistically significant.

## Results

### Patient characteristics

As expected from the matching, the patients from the two groups had similar age, gender and prevalence of diabetes (Table 1). The distribution of primary renal disease was similar except for the percentage of patients with hereditary nephropathies that was significantly higher in the network group.

**Table 1:** Clinical characteristics of patients at inclusion (i.e. 12 months before dialysis initiation)

**Characteristics**	**Whole population**	**Control (n=120)**	**Network (n=40)**	**p**
Age (years)	65.6 ± 14.7	65.6 ± 14.6	65.5 ± 15.4	0.62
Sex (% females)	35.0	35.0	35.0	-
Diabetes (%)	22.5	22.5	22.5	-
Non-smoker (ever) (%)	54.4	55.1	56.4	0.95
BMI (kg/m2)	26.0 ± 5.3	26.2 ± 5.5	25.3 ± 4.4	0.33
eGFR at inclusion (ml/min/1.73m^2^)	14.9 ± 7.3	15.2 ± 8.2	14.2 ± 4.5	0.54
Primary renal disease (%)				
-Glomerulonephritis	26.3	26.7	25.0	0.83
-Diabetic nephropathy	16.9	18.3	12.5	0.1
-Vascular nephropathies	28.1	30.8	20.0	0.15
-Tubulo-interstitial nephropathies	9.4	8.3	12.5	0.45
-Hereditary nephropathies	16.3	12.5	27.5	0.02
-Others	1.9	1.7	2.5	0.74
-Unknown	1.2	1.7	0.0	0.99
Comorbidities (%)				
-Hypertension	95.0	94.2	97.5	0.43
-Heart Failure	17.5	17.5	17.5	-
-Previous MI	10.0	9.2	12.5	0.55
-Previous Stroke or TIA	11.9	14.2	5.0	0.12
-Lower limb vascular disease	25	27.5	17.5	0.16
-Respiratory Failure	16.9	15.8	20.0	0.53
-Cancer	20.0	22.5	12.5	0.16

(*MI*= Myocardial Infarction, *TIA*=Transient Ischemic Attack).

In order to verify the representativeness of the two groups of patients, we compared major characteristics of study patients to those of incident dialysis patients registered during the same period in the French national database of ESRD patients, the REIN (Renal Epidemiology Information Network) Registry. In the whole study group, there were no differences for age and the proportion of women (respectively 65.6±14.7 vs 65.3±15.6 and 35% vs 38%) but there were less diabetic patients (22.5% vs 33.8%; p<0.003) and fewer patients with unknown/others primary renal disease (3.1% vs 12.2%; p<0.0001) than in national database patients.

### Primary endpoints

**Figure 2:**
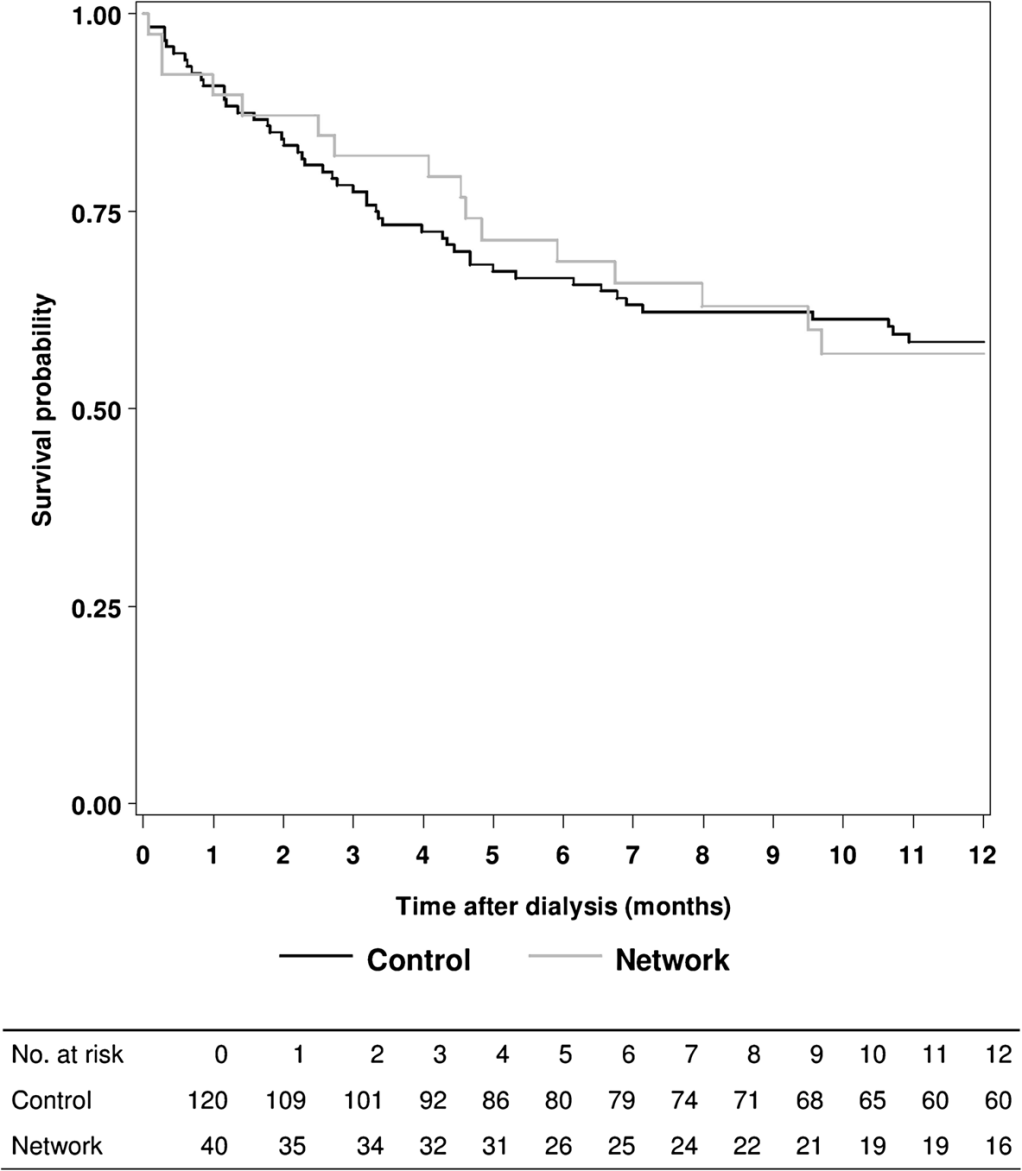
Event-free survival (cardiovascular events or cardiovascular death) using Kaplan-Meier estimates on 160 patients (log-rank p-value=0.9706).

During the first 12 months of dialysis, 16 events (40%) occurred in the network patients whereas 49 events (40.8%) occurred in the control patients (p=0.92, Figure 2). A cardiovascular event occurred in 15 network patients (37.5%) and 46 control patients (38.3%) (p=0.92). The death from cardiovascular cause occurred in 3 network patients (7.5%) and in 8 control patients (6.7%) (p=0.85).

### Secondary endpoints

Five network patients (12.5%) and 17 control patients (14.2%) died during the first year after dialysis start and 22 network patients (55%) and 80 control patients (66.7%) required at least one hospitalization (respectively p=0.79 and p=0.19). The mean number of hospitalizations per patient was significantly higher in the control group (2.0±2.3 vs 1.2±1.6; p=0.03).

During the year before dialysis, 30 network patients (75%) and 100 control patients (83.3%) required at least one hospitalization (p=0.24). The mean number of hospitalizations per patient was significantly higher in the control group (2.3±2.0 vs 1.6±1.7; p=0.04). Hospitalizations needed to start dialysis were more frequent in the control group (32.5% vs 12.5%; p=0.02).

**Figure 3:**
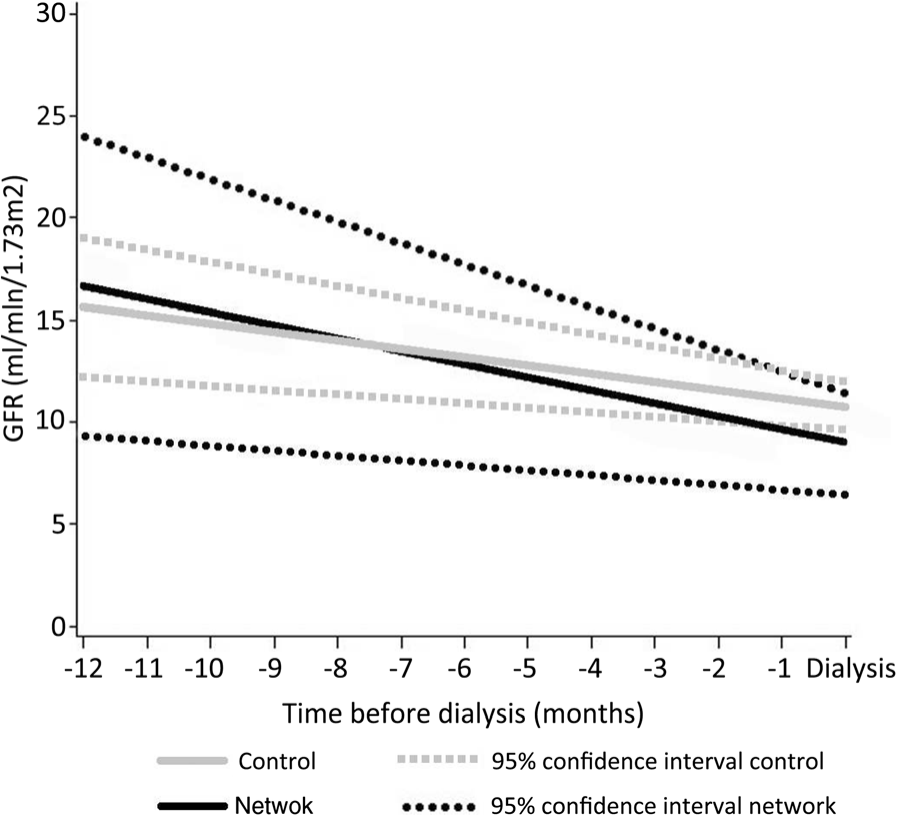
Comparison of the slope of eGFR decline between groups.

The slope of eGFR decline was significantly slower in the network group (7.7±2.5 vs 4.9±1.1 ml/min/1.73m^2^ per year; p=0.04) (Figure 3). The eGFR value at inclusion was similar between the two groups (Table 1) but was significantly higher at dialysis start in network patients (Table 2) which could have spent 4.4 months longer without dialysis before reaching the same eGFR value than control patients (i.e. 9.0 ml/min/1.73m^2^).

**Table 2:** Comparisons of the level of several parameters at the time of the beginning of dialysis

**Parameter**	**Control**	**Network**	**p**
Systolic BP < 130 mmHg (%)	24.5	28.6	0.67
Diastolic BP < 80 mmHg (%)	57.1	62.9	0.53
BP < 130/80 mmHg (%)	20.0	25.7	0.49
BP < 140/90 mmHg (%)	42.9	34.3	0.29
Serum Creatinine (μmol/L)	643 ± 242	535 ± 202	**0.009**
eGFR (ml/min/1.73m^2^)	8.6 ± 3.6	10.3 ± 3.4	**0.012**
Serum Potassium (mmol/L)	4.5 ± 0.8	4.4 ± 0.5	0.82
Serum Urea (mmol/L)	36.0 ± 12.0	31.0 ± 12.0	0.08
Serum Bicarbonates (mmol/L)	21.4 ± 5.0	21.3 ± 5.0	0.97
Serum Calcium (mmol/L)	2.15 ± 0.28	2.24 ± 0.24	0.06
Serum Calcium [2.1-2.6 mmol/l] (%)	60.0	71.8	0.21
Serum Phosphate (mmol/L)	1.95 ± 0.63	1.67 ± 0.58	**0.01**
Serum Phosphate [0.9-1.5 mmol/l] (%)	20.7	46.2	**0.003**
Serum Albumin (g/L)	34.9 ± 6.7	37.1 ± 4.8	0.13
Serum Albumin > 35g/L (%)	55.6	75.9	**0.03**
Haemoglobin (g/L)	105.2 ± 18.1	110.7 ± 15.3	0.1
Haemoglobin [> 110g/L] (%)	37.9	53.9	0.11

During the 12 months before the start of the dialysis, network patients had more nephrology visits (5.6±2.5 vs 4.5±2.5; p=0.02). The proportion of patients without a dietician’s visit was higher in the control group comparatively to the network group (89.2% vs 40%; p<0.001). The mean total number of encounters with health professionals was higher for the network patients (8.8±3.6 vs 6.5±3.7; p=0.003) as was the frequency of measurement of relevant biological parameters (Table 3).

**Table 3:** Comparisons of the frequency of several biological tests in the year before dialysis initiation

**Parameter (mean number of assays by patient)**	**Control**	**Network**	**p**
Serum Creatinine	6.8 ± 2.6	8 ± 2.7	**0.01**
Serum Urea	5.8 ± 2.9	7.3 ± 2.7	**0.006**
Serum Potassium	6.2 ± 2.7	7.8 ± 2.7	**0.002**
Serum Bicarbonate	5.1 ± 2.9	6.5 ± 3	**0.01**
Serum Calcium	5.6 ± 2.9	6.7 ± 2.8	**0.03**
Serum Phosphate	5.3 ± 2.8	6.6 ± 2.9	**0.01**
Serum PTH	1.1 ± 1.2	2.3 ± 2	**< 0.001**
Serum Vitamin D	0.4 ± 0.7	0.6 ± 0.8	0.14
Serum Albumin	1.7 ± 2.2	1.4 ± 1.6	0.52
Lipid profile	1.3 ± 1.5	2.1 ± 1.4	**0.01**
Haemoglobin	6.2 ± 2.9	7.3 ± 2.8	**0.04**
Serum Ferritin	1.8 ± 1.8	2.8 ± 2.3	**0.006**
Proteinuria	1.4 ± 1.6	1.6 ± 1.7	0.48
24h-Urine Sodium	1.1 ± 1.5	1.7 ± 1.9	**0.05**
24h-Urine Urea	0.9 ± 1.5	1.6 ± 1.8	**0.04**

Concerning medications taken by the patients, 151 patients received at least one BP lowering agent (95% vs 92.5% for control and network groups, respectively; p=0.55). Amongst them, 115 received at least one blocker of the RAS (73.7% vs 83.8% for control and network groups, respectively; p=0.18). Sixty-two patients received a beta-blocker (41.7% vs 30% for control and network groups respectively; p=0.2). Fifty-five patients received an antiplatelet agent (33.3% vs 37.5% for control and network groups respectively; p=0.6). One hundred and thirty-five patients received a lipid-lowering drug (82.5% vs 90% for control and network groups respectively; p=0.26). Among the 115 patients receiving at least one medication to correct anaemia, 105 patients received an erythropoiesis stimulating agent (89.7% vs 96.4% for control and network groups respectively; p=0.4), and 52 patients received iron either by oral or iv route (44.8% vs 46.4% for control and network groups respectively; p=0.99). Ninety-one patients from the control group (75.8%) and 36 patients from the network group (90%) received a treatment for the prevention of CKD-mineral bone disease (p=0.06). There was no difference between groups for the prescription of specific components of the treatment (namely calcium salts, vitamin D, or calcimimetics).

Before the first dialysis session, the achievement of treatment goals regarding BP was identical in the two groups (Table 2). The control of serum phosphate and albumin levels was better in the network group (Table 2).

There were some relevant differences regarding pre-dialysis management issues (Table 4).

**Table 4:** Comparisons of several patient conditions between the two groups

**Patient condition**	**Control**	**Network**	**p**
Received information on dialysis (%)	84.2	100	0.99
Information delivered at a nephrology visit (%)	73.3	92.5	**0.02**
Attended education sessions (%)	1.7	57.5	**< 0.001**
Unplanned first dialysis session (%)	36.7	25.0	0.17
First dialysis modality:			
-haemodialysis (%)	71.7	67.5	0.62
-peritoneal dialysis (%)	28.3	32.5	
Use of sustainable dialysis access for first dialysis (AV fistula or catheter) (%)	69.2	82.5	0.1
If haemodialysis, type of first dialysis facility:			
-hospital center (%)	80.2	70.4	0.33
-out-of-hospital unit (%)	12.8	14.8	
-self-dialysis unit (%)	7.0	14.8	
Type of dialysis facility at 12 months:			
-center (in/out hospital) haemodialysis (%)	60.2	53.9	0.71
-self-care haemodialysis (%)	15.1	11.5	
-peritoneal dialysis (%)	24.7	34.6	
Pre-dialysis registration on transplant waiting list (%)	15.0	20.0	0.42
Registration on transplant waiting list within the first year on dialysis (%)	26.5	23.7	0.55

## Discussion

Despite the lack of difference on the primary endpoint, the main finding of our study is that patients treated in a dedicated renal care network were less frequently hospitalized both during the year before and after dialysis start. Moreover, they enter dialysis with better control of serum phosphate, higher proportion of normal serum albumin and received more frequently educations and information’s sessions on CKD and dialysis techniques. Finally, they progressed more slowly towards ESRD. This could have a major impact on the quality of life of the patients, and on the economical burden of renal care [24].

Regarding the impact of MDRC on the early survival of incident dialysis patients, our results differ from previous studies. In a case–control study, Curtis and colleagues showed that exposure to MDRC of CKD patients was associated with a better survival [15]. However, in Curtis et al. Study the mortality rate was rather high likely because of a greater proportion of diabetic patients. Despite the lack of information regarding other comorbidities, it cannot be excluded that patients from the study by Curtis et al. were at higher risk than ours, resulting in an enhanced effect of therapeutic intervention [15]. Similarly, in a cohort study, Goldstein and colleagues found a better survival and fewer hospitalizations in patients followed in MDRC before dialysis, as compared to patients receiving standard care [16]. This difference could be also due to a higher basal risk of death and cardiovascular events, because of a higher proportion of patients with diabetes and/or previous cardiovascular diseases. In line with the hypothesis that the effect of network care was minimized by the recruitment of patients at relatively low risk, the comparison with the whole population of contemporary incident dialysis patients within the REIN Registry showed a lower prevalence of diabetes (22.5% versus 41%) and heart failure (17.5% versus 28.1%) amongst the study patients, resulting in a lower mortality rate during the first year on dialysis (13.8% versus 17%) [3]. In addition, in our study, the rate of use of cardiac and renal protective medications was higher (almost 93% for blockers of RAS) than previously observed [25], and did not differ between the two groups. This may have contributed to the lack of differences on the primary endpoint. The percentage of patients taking an anti-platelet agent was similar between the two groups, close from the figures reported previously [26-28]. Finally, the length of follow-up in our study could have been too short to detect differences on major outcomes. It is noteworthy that Curtis and colleagues found only a slight difference on survival curves after one year of follow up [15] between the MDRC and the control groups.

Network patients required fewer individual hospitalizations before and after the beginning of dialysis, as well as for first dialysis session. There was a trend towards a decrease of unplanned first dialysis and an increase of the proportion of sustainable access used for this first dialysis (Table 4). This occurred despite a higher proportion of patients with usable and sustainable access for first dialysis in the control group comparatively to the mean proportion in the REIN registry (69.2% vs 63%) [3], that suggests a high quality of care for the creation of dialysis access.

The eGFR at dialysis initiation in network patients was similar to that observed in the REIN registry (9.6 ml/min/1,73m^2^) [3], but it was lower in the control group. This difference in average eGFR at dialysis initiation has had probably no impact on the primary outcome and on the one-year mortality rate. Indeed, the IDEAL study showed that there were no difference in the one-year mortality rate between two randomized groups of patients who started dialysis respectively with 9.0 and 7.2 ml/min/1,73m^2^ of average eGFR [29]. Even if the impact of this difference is not known on the occurrence of cardiovascular events during, it seems unlikely that there could be an impact on the primary outcome of our study. So patients from the control group were theoretically more prone to develop metabolic and nutritional disorders, as shown by the lower proportion of patients with normal serum phosphate and albumin levels at dialysis initiation (Table 2). However, some others relevant biological parameters were not different between groups. The decreased rate of hospitalizations is likely related to a lower occurrence of complications, including those requiring unplanned dialysis like electrolytic disorders or acute pulmonary oedema. The incidence of these complications could be positively affected by educational sessions about lifestyle changes and by visits with trained dieticians, which were more frequent in the network patients.

An interesting result of our study is that network patients had a slower decline of renal function (Figure 3), with the dialysis start virtually postponed by more than 4 months. Those results are consistent with previous findings by Devins et al. and more recently by Bayliss et al. [14,30]. Devins and colleagues showed in a randomized controlled trial that a psycho-educational intervention added to usual care allowed delaying dialysis start by 3 months. Educational sessions were also included in our network care and may contribute to the observation of close results between the study by Devins et al. and ours [30]. The results of the observational study by Bayliss and colleagues showed a slow-down of eGFR decrease. This study included patients with a large proportion of diabetes and obesity, a higher baseline eGFR and slower decline than in our study. The control of diabetes and BP was similar in the two groups, suggesting an impact of the MDRC intervention on other progression factors such as dietary habits or use of nephrotoxic medications [14].

The proportion of patients with hereditary diseases was higher in the network group (Table 1). Differences in the distribution of primary nephropathies might have had an influence on the results. However, in advanced stages of CKD, the nature of primary renal disease has only a minor influence on the slope of eGFR decrease [31]. Therefore, the differential progression of CKD is more likely related to the difference in renal care organization.

Our study was designed to assess the effects of coordinated renal care within a distributed network involving healthcare professionals working in the community. At the difference of centralized MDRC in which the services are provided within a same centre [15,16], the network allows the patients to maintain ambulatory encounters with family practitioners. In addition, it provides a biological monitoring closer to CKD management guidelines than usual renal care (Table 2). This could explain the favourable impact of network care on CKD progression. A recent study by Hotu et al. showed that in diabetic and hypertensive CKD patients, a community based model of care leads to a higher decrease in proteinuria and BP than in patients receiving usual care [18]. To our knowledge, this issue was addressed by only one observational study [11]. The authors showed that a distributed network could improve the progression of CKD in patients with stage 3–5 CKD comparatively to usual care. However, in this study, renal follow up was only performed by the GP, without visits with the nephrologist who gave the treatment adaptations remotely [11]. This is quite different from our approach because the absence of direct contact between patients and nephrologist may affect the clinical evolution of the patients.

This study describes for the first time in France the effects of coordinated renal care provided by a dedicated network. Although accurate epidemiological data on the treatment of stages 4–5 CKD are lacking, data from the REIN registry show a trend towards a stabilization of the incidence rate of ESRD over the five last years. However, the prevalence of CVD remains high in incident patients, contributing to their early mortality: French data network REIN showed that the risk of death is highest in the first year after the start of dialysis treatment (17% for the whole population), especially in older patients [3]. In a context of a public social insurance allowing virtually unlimited access to renal caregivers, a way to improve the results of the health system could be to implement new therapeutic strategies based on care coordination through renal care networks. Moreover, the French public health system, as in several countries, is actually subject to an economic pressure because the resources allocated tend to become limited and care cost is becoming more expensive (due to the aging of the population and to several others reasons like incoordination of some provided care). Therefore care network could also help to reduce the costs related to the management of ESRD, which is presently crucial because it is a costly disease.

These results must be interpreted in the light of some limitations of the study. The two main limitations are related to the design of the study. This was an observational matched cohort study and although patients were matched on important confounders, it cannot be excluded that some others confusion parameters may have influenced the results. Another limitation was the difficulty to collect retrospective data from 24h urine measurements that precluded any analysis of the relationship between proteinuria and outcomes. However, the predictive value of proteinuria on cardiovascular events has been mainly assessed in earlier CKD stage patients [2] and it is unclear if the inclusion of proteinuria would have changed the meaning of the results in our late 4–5 CKD stage patients. Finally, one other limitation to mention is that our study was only interested by patients with stages 4–5 CKD. It can be speculated that the results could have been different with patients who wouldn’t have started dialysis (being treated only conservatively) or in patients with less severe CKD (i.e. stage 3 CKD).

## Conclusions

In conclusion, our study shows that a dedicated renal cares network based on a distributed design is effective to decrease the rate of hospitalizations either in the year before and after dialysis initiation. Moreover, it could lower the slope of GFR decrease in patients with advanced CKD. Although no effects of the network were observed concerning the primary endpoint, this could result from a short follow-up period or being related to the CKD stages of the included patients. These results suggest that the management of patients with CKD in a renal care network could improve some of the patient’s outcomes but further studies are needed to confirm this positive effect.

## Abbreviations

CKD: Chronic Kidney Disease; ESRD: End Stage Renal Disease; GP: General Practicionner; MDRC: Multidisciplinary Renal clinics; GFR: Glomerular Filtration Rate; eGFR: Estimated GFR; REIN: Renal Epidemiology Information Network; BP: Blood Pressure; RAS: Renin angiotensin system.

## Competing interests

The authors declare that they have no competing interests.

## Authors’ contributions

The contributions of each author are the following: NR contributed to the data analysis and interpretation, manuscript drafting. ML contributed to the conception and design of the study, data analysis and interpretation, manuscript drafting. CM, SF and CC: contributed to the conception and design of the study, data analysis and interpretation, manuscript revision. EA, JCA, BV, PD, PC, LJ, FV: contributed to the design of the study, data analysis and interpretation. All authors read and approved the final manuscript.

## Pre-publication history

The pre-publication history for this paper can be accessed here:

http://www.biomedcentral.com/1471-2369/14/41/prepub
